# Is There a Relationship Between Cyber-Dependent Crime, Autistic-Like Traits and Autism?

**DOI:** 10.1007/s10803-019-04119-5

**Published:** 2019-07-02

**Authors:** Katy-Louise Payne, Ailsa Russell, Richard Mills, Katie Maras, Dheeraj Rai, Mark Brosnan

**Affiliations:** 1 Department of Psychology, Centre for Applied Autism Research, University of Bath, Bath, BA2 7AY UK; 2 Bristol Medical School, Population Health Sciences, University of Bristol, Bristol, BS8 2BN UK

**Keywords:** Cyber-dependent crime, Digital skills, Autism, Autistic-like traits, Explicit social cognition, Interpersonal support

## Abstract

International law enforcement agencies have reported an apparent preponderance of autistic individuals amongst perpetrators of cyber-dependent crimes, such as hacking or spreading malware (Ledingham and Mills in Adv Autism 1:1–10, 2015). However, no empirical evidence exists to support such a relationship. This is the first study to empirically explore potential relationships between cyber-dependent crime and autism, autistic-like traits, explicit social cognition and perceived interpersonal support. Participants were 290 internet users, 23 of whom self-reported being autistic, who completed an anonymous online survey. Increased risk of committing cyber-dependent crime was associated with higher autistic-like traits. A diagnosis of autism was associated with a decreased risk of committing cyber-dependent crime. Around 40% of the association between autistic-like traits and cyber-dependent crime was mediated by advanced digital skills.

Ledingham and Mills (2015) define cybercrime as “The illegal use of computers and the internet, or crime committed by means of computers and the internet.” Within the legal context (e.g. in the USA, UK, Australia, New Zealand, Germany, the Netherlands and Denmark; Ledingham and Mills 2015), there are two distinct types of cybercrime: (1) cyber-dependent crime, which can only be committed using computers, computer networks or other forms of information communication technology (ICT). These include the creation and spread of malware for financial gain, hacking to steal important personal or industry data and distributed denial of service (DDoS) attacks to cause reputational damage; and (2) cyber-enabled crime such as fraud, which can be conducted online or offline, but online may take place at unprecedented scale and speed (McGuire and Dowling 2013; The National Crime Agency: NCA 2016). In England and Wales, all forms of cybercrime were included in the Office for National Statistics crime estimates for the first time in 2016, which resulted in a near doubling of the crime rate. Cyber-dependent crime specifically represented 20% of UK crime (Office for National Statistics 2017) and in England and Wales in 2018, 976,000 cyber-dependent computer misuse incidents were reported (computer viruses and unauthorised access, including hacking: Office for National Statistics 2019). Furnell et al. (2015) propose that it is more important to understand the factors leading to cyber-dependent incidents and how to prevent them, than to focus on metrics such as specific costs to the global economy. Having interviewed cyber-dependent criminals, the NCA’s intelligence assessment (2017) identified that perpetrators are likely to be teenage males who are unlikely to be involved in traditional crime and also that autism spectrum disorder (ASD, hereafter autism) appears to be more prevalent amongst cyber-dependent criminals than the general populace—though this remains unproven. No socio-demographic bias has yet been identified amongst cyber-dependent offenders or those on the periphery of criminality.

This apparent relationship between cyber-dependent crime and autism is echoed in a survey of six international law enforcement agencies’ (UK; USA; Australia; New Zealand; Germany; the Netherlands; Denmark) experiences and contact with autistic^1^ cybercriminals (Ledingham and Mills 2015), which indicated that some autistic individuals commit cyber-dependent offences. Offences committed included: hacking; creating coding to enable a crime to be committed; creating, deploying or managing a bot or bot-net; and malware (Ledingham and Mills 2015). This was a small-scale study, limiting the generalisability of findings, but it does indicate a presence of autistic offenders within cyber-dependent crime populations, although the link between autism and cyber-dependent crime remains largely speculative as cyber-dependent criminality may be evidenced within a wide range of populations. Further clarification of any relationship between autism and cyber-dependent crime is required before any conclusions can be inferred.

Studies in Asia, Europe, and North America have identified an average prevalence of autism of between 1% and 2% (CDC 2018). Autism is a long-term condition predominately diagnosed in males, characterised by persistent deficits in social communication and interaction coupled with restricted and repetitive patterns of behaviour, interests or activities (American Psychiatric Association 2013; CDC 2018). One possibility is that the anecdotal evidence of apparent autism-like behaviour in cyber-dependent criminals may actually be reflecting people with high levels of autistic-like traits who do not have a diagnosis of autism (Brosnan in press). Autistic-like traits refer to behavioural traits such as social imperviousness, directness in conversation, lack of imagination, affinity for solitude, and difficulty displaying emotions (Gernsbacher et al. 2017). Autistic-like traits are argued to vary continuously across the general population, with studies reporting that autistic groups typically have higher levels of autistic-like traits than non-autistic comparison groups (Baron-Cohen et al. 2001, 2006; Constantino and Todd 2003; Kanne et al. 2012; Plomin et al. 2009; Posserud et al. 2006; Skuse et al. 2009; see also Bölte et al. 2011; Gernsbacher et al. 2017; Ronald and Hoekstra 2011; Ruzich et al. 2015a for meta-analysis). Autistic-like traits are typically assessed through self-report measures such as the 50-item Autism Spectrum Quotient (AQ: Baron-Cohen et al. 2001; see also Baghdadli et al. 2017). Ruzich et al.’s (2015a) meta-analysis of responses to the AQ from almost 7000 non-autistic and 2000 autistic respondents identified that non-autistic males had significantly higher levels of autistic-like traits than non-autistic females, and that autistic people had significantly higher levels of autistic-like traits compared to the non-autistic males (with no sex difference within the autistic sample). A clinical cut-off of a score of 26 on the AQ has been proposed to be suggestive of autism (Woodbury-Smith et al. 2005b), and whilst there are similarities between those with and without a diagnosis of autism who score above the cut-off the AQ, the AQ is not diagnostic. Importantly, there are also *differences* between those with and without a diagnosis of autism who scored above the cut-off (Ashwood et al. 2016; Bralton et al. 2018; Focquaert and Vanneste 2015; Lundqvist and Lindner 2017; see also Frith 2014).

With respect to cyber-dependent crime, some members of both autistic and high autistic-like trait groups will have developed advanced digital skills that are likely to be required to commit cyber-dependent crime. Indeed a specific relationship between ‘autism and the technical mind’ has been previously speculated by Baron-Cohen (2012; see also Wei et al. 2013). Moreover, computer science students and those employed in technology are two of the groups who typically possess higher levels of autistic-like traits (Baron-Cohen et al. 2001; Billington et al. 2007; Ruzich et al. 2015b). These relationships are potentially significant, as cyber-dependent criminal activity requires an advanced level of cyber-related skills (such as proficiency in programming in Java, C/C++, disassemblers, and assembly language and programming knowledge of scripting languages [PHP, Python, Perl, or Shell]; Insights 2018). Thus, there may be an association between autistic-like traits and the potential to develop the advanced digital skills required for cyber-dependent crime.

Assessing the relationship between autistic-like traits and cyber deviancy in a sample of college students, Seigfried-Spellar et al. (2015) found that of 296 university students, 179 (60%) engaged in some form of cyber-deviant behaviour (such as hacking, cyberbullying, identity theft, and virus writing) and the AQ distinguished between those who did and those who did not self-report cyber-deviant behaviour, with higher AQ scores among those reporting cyber-deviant behaviours. The authors also reported that if they used a cut-off score on the AQ of 26 to indicate high levels of autistic-like traits associated with autism, then 7% of the computer non-deviants and 6% of the computer deviants scored in this range. The authors concluded that ‘based on these findings alone, there is no evidence of a significant link between clinical levels of [autism] and computer deviance in the current sample. Nevertheless, the current study did find evidence for computer deviants reporting more autistic-like traits, according to the AQ, compared to computer non-deviants’. However, ‘cyber-*deviant*’ behaviour in Seigfried-Spellar et al.’s study included both cyber-*enabled* crimes such as cyberbullying and identity theft, as well as cyber-*dependent* crimes such as hacking and virus writing. This requires a more nuanced examination as there may be important differences in the relationship between autistic-like traits and cyber-dependent crime compared with cyber-enabled crime.

Cyber-enabled crime is an online variant of traditional crimes (such as fraud) and shares common motivations such as financial gain, whereas the motivations for cyber-dependent crime can be based around a sense of challenge in hacking into a system or enhanced reputation and credibility within hacker communities (NCA 2017). This may be pertinent for the relationship between cyber-*dependent* crime specifically and autism or autistic-like traits, since cyber-dependent criminals typically have not engaged in traditional crime (NCA 2017) and autism has been associated with generally being law abiding and low rates of criminality (Blackmore et al. 2017; Ghaziuddin et al. 1991; Heeramun et al. 2017; Howlin 2007; Murrie et al. 2002; Wing 1981; Woodbury-Smith et al. 2005a, 2006). In addition, several studies have suggested that autistic internet-users can demonstrate a preference for mediating social processes online, such as preferring to use social media over face-to-face interaction to share interests (Brosnan and Gavin 2015; Gillespie-Lynch et al. 2014; van der Aa et al. 2016). This may be significant, as it has been suggested that social relationships developed online are key to progressing into cyber-dependent crime, with forum interaction and reputation development being key drivers of cyber-dependent criminality (NCA 2017).

Finally, failing to appreciate the impact of crime upon others may be a relevant factor, as autism has been argued to reflect a diminished social cognition (e.g., theory of mind, Baron-Cohen et al. 1985). It has been suggested that there are two levels of social cognition; namely, a quicker and less conscious implicit social cognition, and a more conscious, slower and controlled explicit social cognition (Frith and Frith 2008; see also Heyes 2014). Autistic individuals are often not impaired in explicit social cognition, but are reportedly impaired on implicit social cognition (Callenmark et al. 2014; see also Dewey 1991; Frith and Happé 1999). This profile is also reflected in non-social cognition such as reasoning (Brosnan et al. 2016, 2017; Lewton et al. 2018) which may be better characterised as impaired processing of automatic, cognitively efficient heuristics (Brosnan and Ashwin 2018; Happé et al. 2017). Explicit social cognition is therefore a more pertinent measure of the potential to consider the impact of crime upon others.

The aim of the present study was to explore the apparent relationship identified by international law enforcement agencies between autistic-like traits and cyber-dependent crime. To do this, we conducted an online survey exploring autistic-like traits, cyber-related activities (legal and illegal) as well as perceived interpersonal support and explicit theory of mind. Our research question addressed whether higher autistic-like traits, lower explicit theory of mind and lower perceived interpersonal support would increase the risk of committing cyber-dependent crime. We also addressed whether autistic-like traits would be associated with cyber-dependent crime and whether this relationship would be mediated by advanced digital skills. Given the findings associating higher levels of law-abiding behaviour with autism, we also speculated that autism may represent a group of individuals with higher levels of autistic-like traits, but without a higher risk of committing cyber-dependent crime.

## Method

### Participants

The sample comprised 290 participants who reported having not had contact with the criminal justice system. There were 194 male and 96 female participants. Ages ranged from 14 to 74 with a mean age of 24.24 (*SD *= 9.25). Participants were recruited through various channels including the University of Bath participant databases containing computer science students and alumni. In addition, computer science students in local schools were contacted as was the ‘Cyber Security Challenge’—an organisation looking to promote the development of cyber-skilled individuals. Through these channels we aimed to obtain a sample likely to contain participants with advanced digital skills. The exclusion criteria were if participants were under 14 years old, had a significant head injury, untreated epilepsy, or if they had been charged with a cybercrime or received a conviction or caution for cybercriminal activity,^2^ which includes anyone charged and subsequently acquitted of committing a cybercrime. Ethical approval was obtained from the University of Bath Psychology Department Ethics Committee.

### Measures

#### Demographics and IQ

Participants were asked to provide demographic information (age, sex, autism diagnostic status). Participants were asked ‘Have you received a formal diagnosis of Autism Spectrum Disorder (e.g. from a clinician)?’ (yes/no). They were also asked to complete the Ravens Advanced Progressive Matrices Set I (RAPM: Raven 1962) as a brief assessment of non-verbal IQ. Set I consists of 12 matrices, and participants select the correct response out of eight possible responses. Set I is usually used as a practice and screening set for the full test, and it draws on all the intellectual processes sampled on the full test (although it does not extend to the highest levels of complexity) and has been successfully used in previous research exploring thinking and reasoning related to autism and autistic-like traits (Brosnan et al. 2017). This measure has adequate validity and reliability and a reported Cronbach’s alpha of 0.78 (see Chiesi et al. 2011, 2012). Scores could range from 0 to 12.

#### Autistic-Like Traits

The Autism Spectrum Quotient (AQ: Baron-Cohen et al. 2001) is a questionnaire which asks participants to indicate their agreement to 50 statements (e.g., ‘I prefer to do things with others rather than on my own’) using a four-point Likert scale (from ‘definitely agree’ to ‘definitely disagree’). Autistic-like responses are recorded as 1, and baseline (non-autistic) responses are recorded as 0. The AQ is a widely used and recognised screening tool to quantify autistic-like traits. It is recommended that individuals scoring a total score above 32 should be referred for a full ASD diagnostic assessment (Baron-Cohen et al. 2001). More recently, a cut-off of 26 has been proposed (Woodbury-Smith et al. 2005b). The AQ demonstrates good reliability (α. = 75-0.84; Broadbent et al. 2013) and validity (ROC = 0.78; Woodbury-Smith et al. 2005b). Scores range from 0 to 50, with higher scores indicative of greater numbers of autistic-like traits.

#### Explicit Social Cognition

The informal test of social know-how (IToSK; Dewey 1991) is an eight-item test of explicit social cognition. It asks participants to rate how people would judge certain behaviours throughout different scenarios. Judgements are made on a four-point scale from fairly normal behaviour in that situation to shocking behaviour in that situation. This test was selected on the basis that is a less well-known assessment, and is therefore more likely to reflect individual ability rather than practise effects. Scores range from 0 to 58, with lower scores indicative of better social know how (i.e., explicit social cognition). To score this test, data was collected from a sample of an additional 114 individuals to obtain UK norm data. The norm data sample comprised 27 males and 87 females (mean age = 32.79; SD = 16.72; see Callenmark et al. 2014).

#### Perceived Interpersonal Support

The Interpersonal Support Evaluation List-12 (ISEL-12; Cohen et al. 1985) is a 12-item questionnaire in which participants rate how well a statement (e.g., ‘I don’t often get invited to do things with others’) applies to them. Responses are indicated on a four-point scale (from ‘definitely true’ to ‘definitely false’) with half of the items being reverse scored. The questionnaire has good statistical properties demonstrating good internal consistency with values between 0.80 and 0.86 (Merz et al. 2014). Scores range from 0 to 36, with higher scores indicative of greater interpersonal support. The ISEL-12 has been reported to be appropriate for autism-related research (see McConachie et al. 2018).

#### Basic and Advanced Digital Skills (BADS)

Participants responded to 10 basic digital skills and 10 advanced digital skills questions of a five-point scale, with the following instructions: ‘Please indicate how true each of the following 10 statements is about you, at this point in time. There are no right or wrong answers. Please be as honest as you can, and do not miss out any items’. There are 10 questions that ask about basic digital skills followed by 10 questions that ask about advanced digital skills. The 10 basic digital skills items were taken from the operational skills items that was developed as part of the ‘DiSTO: From digital skills to tangible outcomes—improving measures and models of digital engagement’ project, by the London School of Economics and the University of Twente (Helsper et al. 2015). The ten advanced digital skills items were taken from the Null Byte website (‘the aspiring white-hat hacker/security awareness playground’^3^). Scores for each subscale range from 10 to 50, and the items are in Appendix.

#### Cyber-Dependent Crime Questionnaire

The cyber-dependent crime questionnaire was designed for this study to ascertain whether individuals had engaged in cyber-dependent criminal behaviour (yes/no) for each of the eight items listed by the UK’s NCA as constituting cyber-dependent crime.^4^ The eight activities are listed in Appendix. Higher scores are indicative of a greater number of cyber-dependent crimes (from 0 to 8).

## Results

Two-hundred-and-ninety individuals without a conviction or caution for cybercriminal activity took part. Of these, 23 self-reported a diagnosis of autism. Consistent with the self-report diagnosis, this group had higher levels of autistic-like traits than those who did not self-report a diagnosis of autism (30.61, *SD *= 9.35) versus (21.68, *SD *= 8.55), *t*(288) = 4.78, p < 0.001. These means fall above and below a proposed clinical cut-off of 26 (Woodbury-Smith et al. 2005b). Whilst participants had not received a conviction or caution for cybercriminal activity, it was likely that some participants would self–report that they had actually engaged in such behaviour (Seigfried-Spellar et al. reported this for 60% of their participants, see above). Overall, 333 cyber-dependent crimes were reportedly committed by 122 individuals (42%), as highlighted in Table 1.

**Table 1:** Cyber-dependent crimes reported to have been committed by the current sample of 290 individuals without a conviction or caution for cybercrime

Cyber-dependent crime	n	% of all cyber-dependent crimes
Phishing	15	4.50
Web Cam Manager	24	7.21
File Hijacking	43	12.91
Key Logging	46	13.81
Screen Shot Manager	44	13.21
Ad-Clicker	11	3.30
Hacking	87	26.13
DDoS	63	18.92
Total	333	100

The characteristics of the sample by the absence or presence of reported cyber-dependent criminal activity are described in Table 2. Those who had carried out one or more cyber-dependent crime were likely to have a higher AQ score, have greater basic and advanced digital skills and were less likely to report having had an autism diagnosis.

**Table 2:** Descriptive statistics of the sample by the absence or presence of any cyber-dependent criminal activity (n = 290)

	No cyber-dependent criminal activity (n = 168)	One or more cyber-dependent criminal activities (n = 122)	*p*
Age mean (SD)	23.5 (10.1)	25.3 (8.4)	0.097
Male sex (n, %)	119 (70.8%)	75 (61.5%)	0.095
RAPM (mean, SD)	10.3 (1.9)	10.3 (1.7)	0.925
AQ Score (mean, SD)*	21.4 (8.6)	23.9 (9.2)	0.014
IToSK (mean, SD)	8.1 (5.4)	8.8 (4.0)	0.235
ISEL 12 (mean, SD)	24.3 (7.3)	24.1 (8.6)	0.902
Basic Digital Skills (mean, SD)**	49.3 (2.0)	49.8 (1.0)	0.009
Advanced Digital Skills (mean, SD)**	34.3 (12.4)	44.0 (8.2)	0.000
Autism diagnosis (n, %)*	18 (10.7%)	5 (4.1%)	0.040

*p* values derived from t-tests, except for the two binary variables (sex and ASD diagnosis) where Chi square tests were used

*RAPM* Ravens advanced progressive matrices, *AQ* Autism Spectrum Quotient, *IToSK* informal test of social know how, *ISEL* interpersonal support evaluation list

*p < 0.05; **p < 0.01

We used logistic regression to estimate the association between the AQ score and endorsing one or more cyber-dependent criminal activity. We estimated odds ratios with 95% confidence intervals in a crude model without any covariates. We then repeated these analyses adjusting for age, sex and IQ (Model 1) followed by a further adjustment for the presence of an autism diagnosis (Model 2). The results suggest a 4% increased adjusted odds of having carried out at least one cyber-dependent criminal activity with each unit increase in AQ score (see Table 3).

**Table 3:** Logistic regression analysis to assess the association between AQ Total score (exposure) and one or more cyber-dependent criminal activities (outcome)

	Odds Ratio (95% CI)	*p*
Crude OR	1.03 (1.01–1.06)	0.015
Model 1	1.03 (1.00–1.06)	0.025
Model 2	1.04 (1.01–1.07)	0.004

Model 1 adjusted for age, sex, IQ. Model 2 adjusted for age, sex, IQ and an ASD diagnosis

Although the temporal sequencing of the variables was unknown in this cross-sectional study, these variables could be considered to be on the causal path-way between epilepsy and poor mental health Although the temporal sequencing of the variables was unknown in this cross-sectional study, these variables could be considered to be on the causal path-way between epilepsy and poor mental health Although the temporal sequencing of the variables was unknown in this cross-sectional study, these variables could be considered to be on the causal path-way between epilepsy and poor mental health.

Although the temporal sequencing of the variables was unknown in this cross-sectional study, these variables could be considered to be on the causal path-way between epilepsy and poor mental health.

**Fig. 1:**
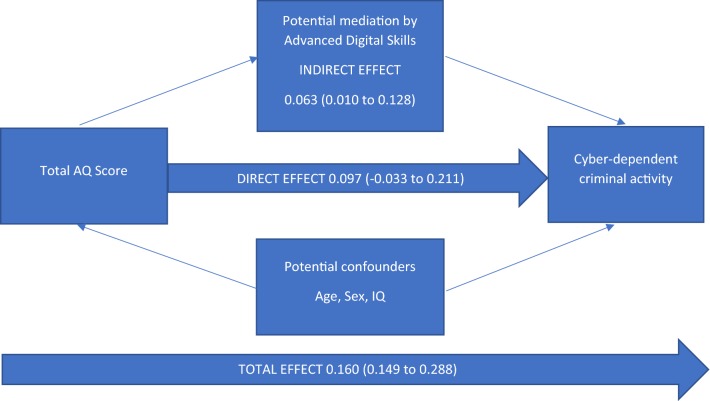
Mediation analysis of the role of advanced digital skills in the association between total AQ scores and cyber-dependent criminal activities. Proportion of total effect mediated by advanced digital skills: 39.4%

Although the temporal sequencing of the variables cannot be assessed in this cross-sectional study, we carried out an exploratory mediation analysis to assess whether the association between the AQ score and cyber-dependent criminal activities would occur via the attainment of advanced digital skills (mediator). To quantify the extent of mediation, we used the user written binary_mediation package in Stata to estimate the direct, indirect and total effects, and the proportion of the total effect mediated. We used bootstrapping with 1000 replications to calculate the bias-corrected confidence intervals for these estimates (see Fig. 1).

### Proportion of Total Effect Mediated by Advanced Digital Skills: 39.4%

The model in Fig. 1 assumes that advanced digital skills lie on the causal pathway between AQ score and cyber-dependent criminal activity. Estimates are beta coefficients with bias corrected 95% confidence intervals. The model is adjusted for age, sex and IQ. The results suggest that a substantial proportion of the association (~ 40% of the total effect) between AQ score and cyber-dependent criminal activity is mediated by advanced digital skills. There was also evidence of mediation by basic digital skills (approximately 23%) but not by perceived interpersonal support or explicit social cognition.

Finally, to explore potential differences in computer-related experiences between those who self-report high levels of autistic-like traits who either do or do not self-report a diagnosis of autism, those with an AQ score of 26 or above were analysed for whether they committed a cyber-dependent crime (or not). Of the 16 with a diagnosis of autism, 3 (19%) had committed a cyber-dependent crime and of the 77 without a diagnosis of autism, 50 (65%) had committed a cyber-dependent crime (χ^2^ = 11.53, *df *= 1, *p *< 0.001). There were no significant differences between participants scoring over 26 on the AQ who did and did not have an autism diagnosis in basic or advanced digital skills, *(t*(91) = 0.51 and1.38 respectively, ns) although the absence of a difference due to lack of statistical power cannot be ruled out.

## Discussion

International law enforcement agencies report an apparent relationship between autism and cyber-dependent crime, although any such link remains unproven (Ledingham and Mills 2015; NCA 2017). This was the first study to empirically explore whether autism, autistic-like traits, explicit social cognition, interpersonal support and digital skills were predictors of cyber-dependent criminality. Whilst higher levels of autistic-like traits were associated with a greater risk of committing cyber-dependent crime, a self-reported diagnosis of autism was associated with a decreased risk of committing cyber-dependent crime. Around 40% of the association between autistic-like traits and cyber-dependent crime was attributable to greater levels of advanced digital skills. Basic digital skills were also found to be a mediator between autistic-like traits and cyber-dependent crime, although they accounted for a smaller proportion of the association than advanced digital skills.

These findings are consistent with the proposal that the apparent association between autism and cyber-dependent crime identified by law enforcement agencies may be reflecting higher levels of autistic-like traits amongst cybercriminals but that this does not necessarily equate to autism being a risk factor for cybercrime. This confusion may well arise because typically, autistic people do report higher levels of autistic-like traits than the general population (Ruzich et al. 2015a). Cyber-dependent crime may therefore represent an area that distinguishes high autistic-trait non-autistic groups from autistic groups, consistent with proposal that people with autism differ qualitatively from non-autistic people who are nevertheless high in autistic-like traits (see Ashwood et al. 2016; Frith 2014). The finding that autistic respondents were less likely to commit cyber-dependent crime is also consistent with literature suggesting that autistic people are generally as law abiding, if not more so, than the general population. Lower levels of criminality are shown, at least for certain types of crime (Blackmore et al. 2017; Cheely et al. 2012; Ghaziuddin et al. 1991; Heeramun et al. 2017; Howlin 2007; King and Murphy 2013; Murrie et al. 2002; Wing 1981; Woodbury-Smith et al. 2005a, 2006; but see, Rava et al. 2017; Tint et al. 2017).

Thus, there is evidence that higher AQ scores are associated with higher levels of cyber-dependent crime regardless of an autism diagnosis. As this association was independent from the autism diagnosis, there may be something about autistic-like traits beyond the diagnostic criteria for autism that relates to cyber-dependent criminal activity. The mediation analysis suggests that an association between autistic-like traits and advanced digital skills may represent a key factor. We cautiously state above that those reporting an autism diagnosis were less likely to report cyber-dependent crime. Cautiously, as this could be for various reasons beyond high AQ and autism being different things, including a diagnosis of autism leading to some protection (e.g., more support leading to less potential criminal behaviour; see Heeramun et al. 2017). Importantly, however, there are potential selection issues in relation to individuals who respond to an invitation to complete an online survey on this topic, thus the possibility of selection bias cannot be ruled out. We do not know how many did not respond to the invitations (and therefore could not identify a response rate, for example) and the apparent protective effect could be a chance finding due to small numbers. Future research using larger samples can address such concerns and until that time the suggestion that autism may be protective should be considered speculative, especially as the data is self-reported and diagnostic status could not be independently verified in the present study.

Previous research has identified higher levels of autistic-like traits being present within scientific disciplines in which computer science students and employees are included (Baron-Cohen et al. 2001; Billington et al. 2007; Ruzich et al. 2015b). This study is the first to specify a direct relationship between higher levels of autistic-like traits and advanced digital skills. In addition to being a pre-requisite for committing cyber-dependent crimes, these skills are essential for the cyber security industry which will have an estimated 3.5 million unfulfilled jobs by 2021 (Morgan 2018). This study suggests that targeting groups high in autistic-like traits would be a beneficial strategy to meet this employment need. Given the employment difficulties that can be faced by members of the autistic community (Buescher et al. 2014; Knapp et al. 2009; see also Gotham et al. 2015; Hendricks 2010; Howlin 2013; Levy and Perry 2011; National Autistic Society 2016; Taylor et al. 2015; Shattuck et al. 2012) and that around 46% of autistic adults who are employed are either over-educated or exceed the skill level needed for the roles they are in Baldwin et al. (2014), targeting the autistic community for cyber security employment may be particularly beneficial.

Notwithstanding the limitations described above, this may be particularly pertinent as this study found that a diagnosis of autism was associated with *reduced* cyber-dependent criminality. This would be consistent with perceptions of autistic strengths of honesty and loyalty (de Schipper et al. 2016)—ideal attributes within employment settings. Importantly, this is not to suggest that all autistic people are good with technology, or that all autistic people should seek employment within cyber security industries (see Milton 2018). Rather, this study highlights that in a particularly challenging employment context, some members of the autistic community may be ideally suited to such employment opportunities and emphasises the need for employers to ensure that their recruitment methods and working environments are autism-friendly and inclusive (see Hedley et al. 2017 for review).

The direct link between autistic-like traits and cyber-dependent crime is also consistent with previous research (Seigfried-Spellar et al. 2015) and may extend to a relationship with cyber-enabled crime (such as online fraud). Seigfried-Spellar et al. (2015) explored relationships between autistic-like traits and cyber-deviancy more broadly defined than cyber-dependent crime. Future research could explore whether the level of autistic-like traits, mediated by advanced digital skills, also relates to cyber-enabled crime, and whether there are any direct effects that are specific to cyber-dependent crime. Seigfried-Spellar et al. (2015) and the present study were both cross-sectional studies. The mediation of advanced digital skills between autistic-like traits and cyber-dependent crime has been assumed in the present study, but this could be best established in longitudinal research. Exploring prison populations to identify if ‘traditional’ crime was related to autistic-like traits found no differences between prisoners and the general population (Underwood et al. 2016), which may suggest that autistic-like traits are associated with cybercrime specifically (that is, cyber-dependent crime and potentially cyber-enabled crime).

Sex, age, non-verbal IQ, explicit social cognition and perceived interpersonal support did not significantly relate to cyber-dependent criminal activity, which serves to highlight the salience of autistic-like traits. A potential limitation is that explicit social cognition was assessed, but not implicit social cognition. Based on the autism literature (Callenmark et al. 2014; Dewey 1991; Frith and Happé 1999), we would not necessarily expect difficulties with explicit social cognition in groups with high autistic-like traits. Implicit social cognition was also assessed by Callenmark et al. using interviews after the IToSK. Such interviews, however, do not readily extend to the online context and future research could explore any role of implicit social cognition in cyber-dependent crime. However, recent accounts of implicit social cognition have questioned whether such a system exists and findings from such measures can better be attributed to general attentional processes (Conway et al. 2017; Heyes 2014; Santiesteban et al. 2014, 2015, 2017).

Future research should also focus on autistic communities as well as those convicted of cyber-dependent and cyber-enabled crimes to further develop our understanding of this area, an important aspect of which is the potential strengths some members of the autistic community can bring to cyber security employment.

## Appendix

### Basic and Advanced Digital Skills (BADS)

1. Not at all true of me.
2. Not very true of me.
3. Neither true nor untrue of me.
4. Mostly true of me.
5. Very true of me.

The 10 basic items were: 1. I know how to open downloaded files; 2. I know how to download/save a photo I found online; 3. I know how to use shortcut keys (e.g. CTRL-C for copy, CTRL-S for save); 4. I know how to open a new tab in my browser; 5. I know how to bookmark a website; 6. I know where to click to go to a different webpage; 7. I know how to complete online forms; 8. I know how to upload files; 9. I know how to adjust privacy settings; 10. I know how to connect to a WIFI network.

The 10 advanced questions were: 11. I understand computer networking (e.g. DHCP, NAT, subnetting); 12. I know how to use Linux; 13. I know how to use a sniffer/protocol analyser (e.g. Wireshark, Tcpdump); 14. I know how to use virtualization software packages (e.g. VirtualBox or VMWare workstation); 15. I understand security concepts and technologies (e.g. public key infrastructure, secure sockets layer, etc.); 16. I understand how encryption algorithms work (e.g. WEP, WPA, WPA2); 17. I know how to use one of the scripting languages including the BASH shell (e.g. Perl, Python, Ruby); 18. I understand how databases work (e.g. SQL language); 19. I know how to build my own website; 20. I understand details of TCP/IP protocol stack and fields.

### Cyber-Dependent Crime Questionnaire

The 8 items were: 1. Phishing (sending bogus emails asking for security information and personal details); 2. Webcam managing (taking over someone’s webcam); 3. File hijacking (hijacking someone’s files without their permission); 4. Keylogging (recording what someone types on their keyboard); 5. Screenshot managing (taking screenshots of someone’s computer screen); 6. Employing ad clicking (directing someone’s computer to click on a specific link); 7. Hacking (accessing computer systems); and 8. Launching distributed denial of service (DDoS) attacks (targeting a computer system to prevent it working).

## Footnotes

1 Kenny et al.’s (2016) survey of the autistic community and related professionals identified that many autistic adults prefer to be referred to as autistic rather than as ‘a person with autism’, which we shall reflect in this paper.

2 Targeting cybercriminals within the criminal justice system requires additional ethical procedures which is planned for future research with the National Crime Agency.

3 http://null-byte.wonderhowto.com/how-to/essential-skills-becoming-master-hacker-0154509/.

4 http://www.nationalcrimeagency.gov.uk/crime-threats/cyber-crime.
